# Promoter hypermethylation of the *SFRP2 *gene is a high-frequent alteration and tumor-specific epigenetic marker in human breast cancer

**DOI:** 10.1186/1476-4598-7-83

**Published:** 2008-11-06

**Authors:** Jürgen Veeck, Erik Noetzel, Nuran Bektas, Edgar Jost, Arndt Hartmann, Ruth Knüchel, Edgar Dahl

**Affiliations:** 1 Molecular Oncology Group, Institute of Pathology, University Hospital of the RWTH Aachen, Pauwelsstrasse 30, 52074 Aachen, Germany; 2 Department of Internal Medicine IV (Hematology and Oncology), University Hospital of the RWTH Aachen, Pauwelsstrasse 30, 52074 Aachen, Germany; 3 Department of Pathology, University of Erlangen, Krankenhausstrasse 12, 91054 Erlangen, Germany

## Abstract

**Background:**

We have previously reported that expression of the Wnt antagonist genes *SFRP1 *and *SFRP5 *is frequently silenced by promoter hypermethylation in breast cancer. SFRP2 is a further Wnt inhibitor whose expression was recently found being downregulated in various malignancies. Here we investigated whether SFRP2 is also implicated in human breast cancer, and if so whether *SFRP2 *promoter methylation might serve as a potential tumor biomarker.

**Methods:**

We analyzed *SFRP2 *mRNA expression and *SFRP2 *promoter methylation in 10 breast cell lines, 199 primary breast carcinomas, 20 matched normal breast tissues and 17 cancer-unrelated normal breast tissues using RT-PCR, realtime PCR, methylation-specific PCR and Pyrosequencing, respectively. SFRP2 protein expression was assessed by immunohistochemistry on a tissue microarray. Proliferation assays after transfection with an *SFRP2 *expression vector were performed with mammary MCF10A cells. Statistical evaluations were accomplished with SPSS 14.0 software.

**Results:**

Of the cancerous breast cell lines, 7/8 (88%) lacked *SFRP2 *mRNA expression due to *SFRP2 *promoter methylation (*P *< 0.001). *SFRP2 *expression was substantially restored in most breast cell lines after treatment with 5-aza-2'-deoxycytidine and trichostatin A. In primary breast carcinomas SFRP2 protein expression was strongly reduced in 93 of 125 specimens (74%). *SFRP2 *promoter methylation was detected in 165/199 primary carcinomas (83%) whereas all cancer-related and unrelated normal breast tissues were not affected by *SFRP2 *methylation. *SFRP2 *methylation was not associated with clinicopathological factors or clinical patient outcome. However, loss of SFRP2 protein expression showed a weak association with unfavorable patient overall survival (*P *= 0.071). Forced expression of *SFRP2 *in mammary MCF10A cells substantially inhibited proliferation rates (*P *= 0.045).

**Conclusion:**

The *SFRP2 *gene is a high-frequent target of epigenetic inactivation in human breast cancer. Its methylation leads to abrogation of *SFRP2 *expression, conferring a growth advantage to epithelial mammary cells. This altogether supports a tumor suppressive function of *SFRP2*. Although clinical patient outcome was not associated with *SFRP2 *methylation, the high frequency of this epimutation and its putative specificity to neoplastic cells may qualify *SFRP2 *promoter methylation as a potential candidate screening marker helping to improve early breast cancer detection.

## Background

Aberrant promoter methylation leading to functional inactivation of tumor suppressor genes is a well recognized mechanism capable of driving carcinogenesis [1,2]. In human breast cancer numerous genes have been identified with abolished expression due to 5'-cytosine methylation within their gene promoter (recently reviewed in [3]). Typically those genes affect important aspects of normal growth control, like cell cycle regulation (p16^INK4a^) [4], steroid receptor biology (estrogen receptor-α) [5], cell adhesion (E-cadherin) [6], apoptosis (death-associated protein (DAP) kinase-1) [7] or extracellular matrix integrity (ITIH5) [8], all of which confers, in case of expression loss, growth advantages to neoplastic cells. Importantly, the observation that DNA methylation of the same gene may occur both in premalignant lesions, such as atypical hyperplasia of the breast, and in carcinoma [9] suggests that DNA methylation might serve as ideal biomarker for early cancer detection or patient risk assessment in clinical oncology [10]. Thus, identification and validation of epigenetically silenced cancer-related genes is of critical importance in the search of novel tumor biomarkers.

Secreted frizzled-related proteins (SFRPs) constitute a family of extracellular Wnt signaling antagonists, of which five members (SFRP1-5) have been identified to date [11]. SFRPs sequester Wnt molecules at the cell surface membrane [12] and by this are recognized as sensitive regulators of the canonical Wnt signaling pathway [13]. Aberrant activation of Wnt signaling has been associated with the pathogenesis of virtually all human cancers (reviewed in [14]). In breast tumor tissues, activated Wnt signaling has been repeatedly observed as determined by nuclear and cytoplasmic accumulation of β-catenin [15-18], arguing for a disrupted equilibrium between Wnt and SFRP expression in this tumor type. In line with this, previous studies have shown that expression of SFRP genes is commonly silenced by promoter methylation in human cancers [19-26]. In breast cancer, *SFRP1 *and *SFRP5 *have been identified as targets of aberrant epigenetic inactivation to date, and either promoter methylation was found to be associated with unfavorable patient prognosis [27,28]. *SFRP2 *has been previously identified as epigenetic target in other tumor entities, such as colon [29], oesophagus [30], bladder [31], stomach [23,24], liver [25] and lung cancer [32]. Interestingly, in all tumor entities *SFRP2 *methylation was detected with a high frequency of > 50% of cancer patients, ranging from 52% in lung cancer to 96% in gastric cancer, which suggests that *SFRP2 *methylation might potentially be useful as a ubiquitous pan-tumor marker in cancerous tissues, and possibly also in body fluids. Consequently, Urakami *et al*. [22] demonstrated that of all investigated SFRPs only *SFRP2 *methylation proved to be a valuable independent prediction factor for bladder cancer in urine samples. At the same time, *SFRP2 *methylation was found to occur high-frequent in colon cancer (83–90%) [33,19], which may have forced the establishment of *SFRP2 *methylation as a promising sensitive screening marker for the stool-based detection of colorectal cancer and premalignant lesions [34-36].

Very recently, Suzuki *et al*. [37] reported about *SFRP2 *methylation in human breast cancer, and their study demonstrated an inhibitory effect of SFRP2 on canonical Wnt signaling in breast cancer cell lines. However, *SFRP2 *expression analyses in normal and breast carcinoma tissues as well as patient survival analysis in relation to *SFRP2 *methylation were not addressed. Our approach was to investigate *SFRP2 *expression and promoter methylation in breast cell lines, primary breast carcinomas and normal breast tissues, followed by comprehensive statistical correlation analysis with clinicopathological factors and patient survival. We also investigated a functional role of *SFRP2 *in a breast cell line with regard to growth behaviour. In summary, our data confirm that the *SFRP2 *gene is high-frequently inactivated by promoter methylation in human breast cancer and that loss of *SFRP2 *expression confers a growth advantage to mammary epithelial cells. In addition, we provide evidence that SFRP2 protein expression is commonly reduced in breast cancer and that *SFRP2 *methylation might be a potential biomarker useful for early detection of this disease.

## Methods

### Cryoconserved clinical materials

According to a multi-center study design, 20 matched tumor/macroscopically normal samples of breast cancer patients (median patient age: 67 years; range 48–86 years) and 179 unmatched breast carcinomas (median patient age: 57 years; range 28–96 years) were obtained from patients treated by primary surgery for breast cancer at the Departments of Gynecology at the University Hospitals of Aachen, Jena, Regensburg and Düsseldorf, Germany. None of the patients had received neo-adjuvant chemotherapy. Inclusion criterion for ipsilateral normal breast tissue was a distance of > 2 cm to the carcinoma margin. All patients gave informed consent for retention and analysis of their tissue for research purposes and the Institutional Review Boards of the participating centers approved the study. The selection of cases was based on availability of tissue. Cases were not stratified for any known preoperative or pathological prognostic factor. Tumor histology was determined according to the criteria of the WHO (2003), while disease stage was assessed according to UICC [38]. Tumors were graded according to Bloom and Richardson, as modified by Elston and Ellis [39]. Hormone receptor positivity was defined as an immunoreactivity score (IRS) ≥ 3 [40]. For 136 patients follow-up data were available with a median time of 64 months (range 1–174 months). Tumor material was immediately snap-frozen in liquid nitrogen after surgery. Hematoxylin/Eosin-stained sections were prepared for assessing the percentage of tumor cells; only samples with greater than 70% tumor cells were selected for analysis. Samples were dissolved in lysis buffer followed by DNA isolation using the QIAamp DNA Mini Kit (Qiagen, Hilden, Germany) according to the manufacturer's recommendations. For patient characteristics see additional file 1.

### Formalin-fixed, paraffin-embedded (FFPE) clinical material

A total of 17 archival FFPE normal breast tissues were obtained from the Institute of Pathology, University Hospital of the RWTH Aachen, Germany. These patients had undergone breast reduction surgery without the condition of cancer. The median age in the cancer-unrelated normal breast tissue set was 33 years (range 22–61 years). Per sample, five consecutive sections (each 10 μm) were deparaffinized and rehydrated in a decreasing alcohol series prior to DNA extraction by use of the QIAamp DNA Mini Kit.

SFRP2 protein expression was assessed using a tissue microarray (TMA) consisting of 125 breast carcinomas, four ductal carcinomas *in situ *(DCIS) and 10 normal breast tissues that have been described previously [41]. The TMA contained one tissue core from non-selected, FFPE primary breast carcinoma specimens diagnosed between 1994 and 2002 at the Institute of Pathology, University of Regensburg, Germany. Histological, all tumors were graded according to Bloom and Richardson, as modified by Elston and Ellis [39]. Clinical follow-up data were available for 124 breast cancer patients with a median follow-up period of 80 months (range 5–114 months). All patients gave informed consent for retention and analysis of their tissue for research purposes and the Institutional Review Board of the participating centers approved the study.

### Immunohistochemistry

The TMA was subjected to immunostaining using the K5007 Kit (DAKO, Hamburg, Germany) following the manufacturer's instructions. Antigen retrieval was performed by pretreatment in citrate buffer (pH 7) in a microwave oven (20 min, 200 W). Samples were incubated for 30 min with the primary SFRP2 antibody (rabbit polyclonal IgG; H-140; 1:75; Santa Cruz Biotechnology, Santa Cruz, CA), washed, and incubated for 10 min with the secondary antibody (biotinylated polylink; DAKO). Diaminobenzidin (DAKO) was used for antibody detection. In negative controls the primary antibody was omitted. An experienced breast cancer pathologist (N.B.) scored the immunohistochemical staining according to the scoring system suggested by Remmele and Stegner [40]. Feasibility of the antibody for immunohistochemical analysis of breast tissue has been previously demonstrated *e.g*. by Lee *et al*. [42].

### Cell lines

The benign cell lines HMEC and MCF10A as well as the cancerous breast cell lines BT20, BT474, Hs578T, MCF7, MDA-MB-231, MDA-MB-453, SKBR3, T47D and ZR75-1 were obtained from the American Type Culture Collection (Rockville, MA) and cultured as recommended by the vendor.

### Reverse transcription (RT-) PCR and semi-quantitative realtime PCR

RNA isolation, RT-PCR and SYBR Green I realtime PCR (Roche Diagnostics, Mannheim, Germany) were performed as described elsewhere [27]. Quality of cDNA was checked after each preparation by standard RT-PCR using glyceraldehyde-3-phosphate-dehydrogenase (*GAPDH*) primers that yield an amplification product of 510 bp. To ensure experiment accuracy, all quantitative measurements were performed in triplicate. Intron-spanning primer sequences and cycle conditions are given in additional file 2.

### Sodium bisulfite-modification and methylation-specific PCR (MSP)

Of the genomic DNA, 1 μg was bisulfite-modified using the EZ DNA Methylation Kit (Zymo Research, Orange, CA) according to the manufacturer's recommendations. The final precipitate was eluted in 20 μl TRIS buffer (10 mM). For MSP, one μl of modified DNA was amplified using MSP primers (see additional file 2) that specifically recognized either the unmethylated or methylated gene promoter sequence after bisulfite-conversion. Each primer pair mapped to nine cytosine-phosphate-guanine dinucleotide (CpG) sites in order to specifically discriminate between methylated and non-methylated DNA. Further 11 non-CpG cytosines within the primer pair specific for methylated DNA and 13 non-CpG cytosines within the primer pair specific for non-methylated DNA guaranteed unequivocal amplification of bisulfite-converted DNA. Primers defined an amplicon between +19 and +163 relative to the transcription start site (+1) of the *SFRP2 *gene. Reaction volumes of 25 μl contained 1 × MSP-buffer [44], 400 nM each of methylation and non-methylation-specific primers, respectively, and 1.25 mM of dNTPs. One drop of mineral oil was added to the reaction tube. The PCR was initiated as "Hot Start" PCR at 94°C and held at 80°C before the addition of 1.25 units *Taq *DNA polymerase (Promega, Madison, WI). Cycle conditions were: 95°C for 5 min, 35 cycles of 95°C for 30 s, 60°C for 30 s, 72°C for 40 s and a final extension at 72°C for 5 min. Blood lymphocyte DNA from a healthy donor was bisulfite-modified to serve as a control for the unmethylated promoter sequence [45], DNA from the cancerous breast cell line BT20 served as control for methylated alleles. Amplification products were visualized on 3% low range ultra agarose gel (Bio-Rad Laboratories, Hercules, CA) containing ethidium bromide and illuminated under ultraviolet (UV) light.

### Pyrosequencing

Quantitative Pyrosequencing of a *SFRP2 *promoter fragment was performed by use of a Pyromark ID device, PyroGoldSQA Reagent Kit and Pyro Q-CpG software (Biotage, Uppsala, Sweden). Initially, a 291 bp fragment of the *SFRP2 *promoter (relative position -28 to +263), covering the hybridization sites for MSP primers, was amplified with degenerate primers irrespective of the methylation status, which assures unbiased DNA amplification. To enable single strand preparation the reverse primer was 5'-biotinylated. Reaction volumes of 50 μl contained 1 × Go*Taq *buffer, 2.5 units Go*Taq *polymerase (Promega), 2.5 mM of MgCl_2_, 400 nM of primers, 500 nM of each dNTP, and 3 μl of bisulfite-converted DNA as template. Reactions were initiated as "Hot Start" PCR at 95°C for 3 min and held at 80°C before addition of *Taq *polymerase. Cycle conditions were: 94°C for 3 min, 50 cycles of 94°C for 15 sec, 58°C for 30 sec, 72°C for 30 sec, and a final extension step at 72°C for 5 min. PCR was carried out in a PTC-200 cycler (Bio-Rad, formerly MJ Research, Hercules, CA). Prior to sequencing, aliquots of the amplificate were analyzed on a 2% agarose gel containing ethidium bromide under UV light. Single strand separation of the remaining amplificate (40 μl) was performed with a PyroMark Vacuum Prep Workstation (Biotage). Amplificate was immobilized to Streptavidin-Sepharose HP beads (Amersham Biosciences, Uppsala, Sweden), washed, denatured and the biotinylated strands were released into 40 μl of annealing buffer containing 400 nM of forward sequencing primer. Sequencing started with position +3 (relative to the TSS) and was continued to position +167, covering a total of 22 sequential CpG sites. The following sequence represents the *SFRP2 *promoter sequence that was analyzed by pyrosequencing: AYGGTTTATTTTGTTTTTTYGGGTYGGAGT TTTTYGGAGTTGYGYGYGGGTT TGTAGTGTTTYGTTYGYGTTGTTTTTTYGGTGTTTYGTTTTTTYGYGTT TTAGTYGTYGGTTGTT AGTTTTTYGGGGTTTYGAGTYGTATTTAGYGAAGAGAGYGGGTTYGG. 

Universal bisulfite-converted polymethylated and unmethylated DNA (Epi Tect Control DNA Set; Qiagen, Hilden, Germany) served as technical controls for *SFRP2 *methylation and non-methylation, respectively. Pyrosequencing primers are available on request.

### 5-aza-2'-deoxycytidine (DAC) and trichostatin A (TSA) treatment

We plated cells at 3 × 10^4 ^cells/cm^2 ^in a six-well plate on day 0. The demethylating agent DAC (Sigma-Aldrich, Deisenheim, Germany) was added to a final concentration of 1 μM in fresh medium on days 1, 2 and 3. Additionally, 300 nM TSA (Sigma-Aldrich) was added on day 3. Cells were harvested on day 4 for RNA and DNA extraction. Control cells were incubated without the addition of DAC or TSA and fresh medium was also supplied on days 1, 2 and 3.

### Transient transfection

Cells were seeded at a density of 3 × 10^4 ^cells/cm^2 ^and transfected 24 hours after incubation with 100 ng/cm^2 ^of plasmid DNA in the following manner: 100 ng of empty pCMV-hemagglutinin (HA) vector control (Clontech, Heidelberg, Germany), or 50 ng of pCMV-HA + 50 ng pCMV-HA/*SFRP2*, or 50 ng of pCMV-HA + 50 ng pcDNA3.1-HisA/*WNT1*, or 50 ng pCMV-HA/*SFRP2 *+ 50 ng pcDNA3.1-HisA/*WNT1 *[19] applying the FuGENE 6 transfection system (Roche Diagnostics) and a 3:1 transfection ratio according to the manufacturer's instructions.

### Proliferation assays

MCF10A cells were transfected in 96-well plates as described above and an XTT-proliferation assay (Roche Diagnostics) was performed on day 0, 1, 2 and 3 after transfection by determining the optical density of the supernatants at 480 nm minus the optical density of the supernatants at 690 nm. To enhance experimental accuracy, six replicas were seeded. For the colony formation assay cells were transfected accordingly in six-well plates and kept for three weeks under selective force of the antibiotic G418 (700 μg/ml) (Invitrogen, Carlsbad, CA). After incubation, colonies were washed with phosphate-buffered saline, fixed and stained for 30 minutes (0.25% crystal violet in 10% formalin/80% methanol), washed three times with distilled water and photographed.

### Statistical methods

Statistical analyses were completed using the software package SPSS, version 14.0 (SPSS Inc., Chicago, IL). Differences were considered significant when *P*-values were below 0.05. A two-sided non-parametric Mann-Whitney U-test and a paired student's t-test were performed to analyze differences in expression levels. Associations between metrical variables were determined by a linear regression analysis. To study statistical associations between clinicopathological factors and SFRP2 expression or *SFRP2 *promoter methylation status contingency-tables and two-sided Fisher's exact tests were accomplished. Survival curves were calculated using the Kaplan-Meier method, with significance evaluated by two-sided log-rank statistics. Overall survival (OS) (n = 136 for MSP samples) was measured from the day of surgery until tumor-related death (20.6%, n = 28) and was censored for patients alive at last contact (69.1%, n = 94), in case of death unrelated to the tumor (3.7%, n = 5) or when the death cause was unknown (6.6%, n = 9). Disease-free survival (DFS) (n = 136 for MSP samples) was measured from surgery until local or distant relapse (36.8%, n = 50) and censored for patients alive without evidence of relapse at the last follow-up (63.2%, n = 86).

## Results

### Expression of *SFRP2 *mRNA is reduced in breast cancer cell lines

**Figure 1:**
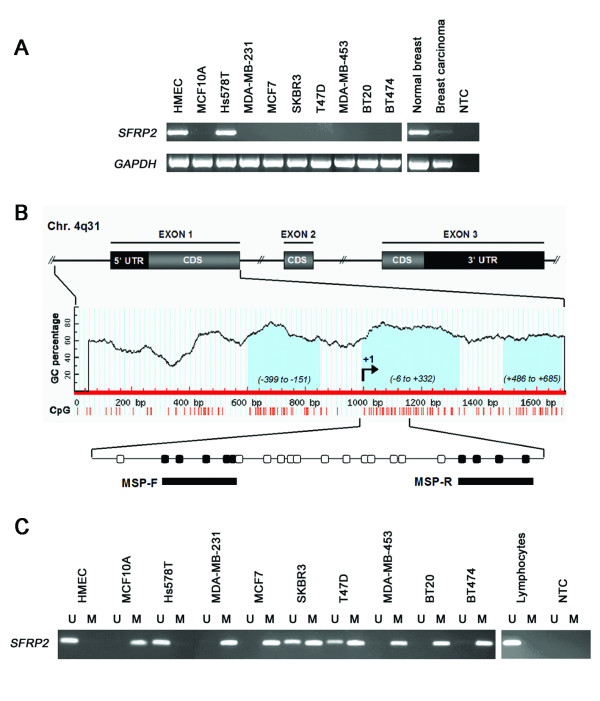
***SFRP2 *expression and promoter methylation in breast cancer cell lines**. (A) *SFRP2 *mRNA expression in benign and malignant cell lines was determined by RT-PCR. All but one malignant cell line (Hs578T) completely lacked *SFRP2 *mRNA expression. Of the two benign breast cell lines (MCF10A and HMEC) only HMEC cells were found to express abundant *SFRP2 *mRNA. In a commercially available human normal breast tissue cDNA *SFRP2 *expression was clearly detectable, while expression was strongly reduced in a corresponding cDNA of human malignant breast tissue. (B) Genomic structure of the human *SFRP2 *gene on chromosome 4q31. Bioinformatic analysis revealed three CGIs (light blue), located within the *SFRP2 *promoter (left CGI), the 5' untranslated region (UTR; central CGI) and the coding sequence (CDS; right CGI). Methylation of the central CGI was explored by MSP. Circles indicate CpG sites; filled circles represent MSP forward (MSP-F) and reverse (MSP-R) primer hybridization sites. Indicated positions are related to the transcription start site (+1) initiating the 5'-UTR. (C) MSP was performed with bisulfite-treated DNA from the same breast cell lines as in A. DNA bands in lanes labeled with U indicate PCR products amplified with primers recognizing unmethylated *SFRP2 *promoter sequence. DNA bands in lanes labeled with M represent amplification products with methylation-specific primers. Five out of eight mammary tumor cell lines exhibit complete promoter methylation (MDA-MB-231, MCF7, MDA-MB-453, BT20 and BT474), two cell lines show partial *SFRP2 *methylation (SKBR3 and T47D). In Hs578T, only unmethylated *SFRP2 *promoter sequence could be detected, like it was also found in benign HMEC cells. In addition, lymphocyte DNA from a healthy donor did not reveal *SFRP2 *methylation. *GAPDH *served as cDNA loading control; NTC represents the no template control.

Initiating our analysis, we used the RT-PCR technique to evaluate whether *SFRP2 *mRNA is differentially expressed in human breast cell lines. *SFRP2 *mRNA expression was detectable in the benign cell line HMEC, whilst its expression was absent in the cancerous cell lines MDA-MB-231, MCF7, SKBR3, T47D, MDA-MB-453, BT20 and BT474 (Figure 1A). Of the cancerous cell lines only Hs578T exhibited strong *SFRP2 *expression. However, *SFRP2 *expression was also absent in the benign cell line MCF10A. Additionally, in a commercially available cDNA of human normal breast tissue (Clontech, Heidelberg, Germany) *SFRP2 *was strongly expressed while its expression was substantially reduced in Clontech's cDNA of primary breast carcinoma.

### Methylation of the *SFRP2 *promoter in breast cancer cell lines

Analysis of the *SFRP2 *gene promoter on chromosome 4q31 [46] using the genomic DNA information contained in Ensembl contig ENSG00000145423 [47] revealed a CpG island (CGI) between base position -818 to +743 relative to the expected transcription start site (+1), according to the CGI definition of Takai and Jones [48]. Further exploration of this CGI using Methprimer software [49] identified three regions of particularly high CpG density (-399 to -151, -6 to +332, and +486 to +685) (Figure 1B). Since sequence integrity of the first *SFRP2 *exon was demonstrated to be most essential for efficient RNA transcription in a luciferase promoter assay [24] we chose the central CGI (-6 to +332) for subsequent methylation analysis by application of the highly specific MSP primers described by Suzuki *et al*. [29] and others [24,37]. First, we assessed *SFRP2 *promoter methylation in eight cancerous and two non-cancerous cell lines. Six of the analyzed cell lines (MCF10A, MDA-MB-231, MCF7, MDA-MB-453, BT20 and BT474) exhibited a methylated *SFRP2 *promoter sequence in the analyzed region (Figure 1C). Two cell lines (SKBR3 and T47D) showed partial promoter methylation, since a mixture of unmethylated and methylated DNA sequence could be detected in the same sample. One malignant cell line (Hs578T) and benign HMEC cells revealed solely unmethylated *SFRP2 *promoter sequence. This result correlates with the above described finding that Hs578T and HMEC cells exhibited strong *SFRP2 *mRNA expression whereas in all cell lines with aberrant methylation *SFRP2 *mRNA expression was absent. Interestingly, *SFRP2 *mRNA was not expressed at detectable levels from unmethyated alleles in SKBR3 and T47D, which may be due to repressing mechanisms that are codominant to the effect of promoter methylation in these cells.

### Re-expression of *SFRP2 *mRNA after *in vitro *DNA demethylation

**Figure 2:**
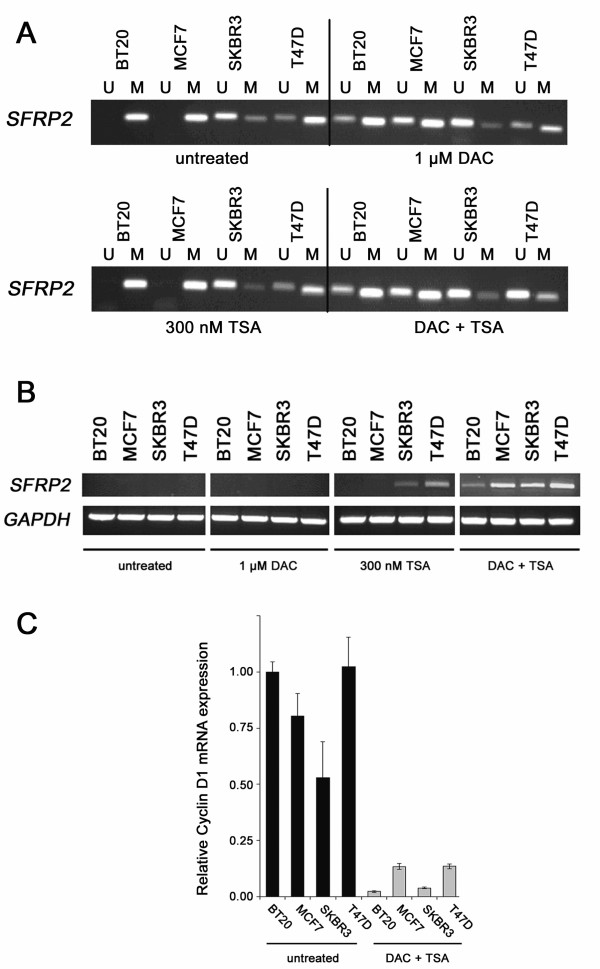
**Global demethylation and histone acetylation restores *SFRP2 *expression**. (A) MSP of four malignant cell lines was performed with DNA from either untreated cells, or after treatment with 1 μM DAC, or after treatment with 300 nM TSA, or after a combined treatment applying both drugs. In three cell lines (BT20, MCF7, T47D) a promoter demethylating effect could be visually detected, since signals indicative of unmethylated *SFRP2 *promoter arise (BT20, MCF7) or become enhanced (T47D) after the combined treatment. In T47D, DAC alone had no detectable demethylating effect on the *SFRP2 *promoter. (B) Expression of *SFRP2 *mRNA before treatment, or after treatment with 1 μM DAC, or after treatment with 300 nM TSA, or after a combined treatment applying both drugs. Treatment with DAC alone was not able to induce *SFRP2 *expression in all cell lines, in contrast to TSA which induced expression in two out of four cell lines (SKBR3 and T47D) previously showing partial *SFRP2 *methylation. However, only combined promoter demethylation and histone reacetylation leads to strong induction of *SFRP2 *mRNA expression in all cell lines. *GAPDH *served as cDNA loading control. (C) Suppression of Cyclin D1 mRNA expression after global DNA demethylation of breast cancer cell lines as determined by realtime PCR. Untreated tumor cells (black bars) and cells treated with DAC/TSA (grey bars) show significantly different expression levels of Cyclin D1 mRNA (*P *= 0.029, two-sided Mann-Whitney U-test). Expression level of each sample is normalized to its *GAPDH *expression and related to untreated BT20 cells (set to 1).

We next asked whether *SFRP2 *promoter methylation is functionally associated with loss of *SFRP2 *mRNA expression in breast cell lines. To address this question we treated four representative breast cancer cell lines for four days with 1 μM of the methyltransferase inhibitor DAC and one day with 300 nM of the histone deacetylase inhibitor TSA. As seen in Figure 2A, in three cell lines (BT20, MCF7 and T47D) the DAC/TSA treatment resulted in a clear increase of unmethylated *SFRP2 *promoter sequence, only in SKBR3 cells there was no visible difference detectable. Interestingly, BT20 and MCF7 showed demethylation after the addition of DAC alone, whereas in T47D cells such effect was only achieved by the combination of DAC and TSA. Before the treatment, none of the cell lines showed detectable *SFRP2 *mRNA expression (Figure 2B). The treatment with 1 μM DAC alone did not reverse *SFRP2 *gene repression in these cells. TSA, in contrast, was able to induce some *SFRP2 *expression in SKBR3 and T47D cells. Importantly, only the combination of both drugs substantially restored *SFRP2 *mRNA expression in all analyzed cell lines.

To further prove that the demethylating treatment did not result in unspecific upregulation of gene expression, we determined the expression of the growth promoting gene cyclin D1, which is a direct read-out gene of active Wnt signaling [50] and whose expression is commonly elevated in breast cancer [51]. Using realtime PCR we observed that cyclin D1 mRNA expression was significantly reduced (*P *= 0.029, two-tailed Mann-Whitney U-test) after the demethylation treatment (43-fold in BT20, 14-fold in SKBR3, 8-fold in T47D and 6-fold in MCF7, Figure 2C), suggesting that inhibitors of proliferation, such as SFRPs which are downregulated in breast cancer cell lines, have been reactivated and were able to block Wnt signaling in these cells.

### Methylation of the *SFRP2 *promoter in primary breast carcinoma and normal breast tissue

**Figure 3:**
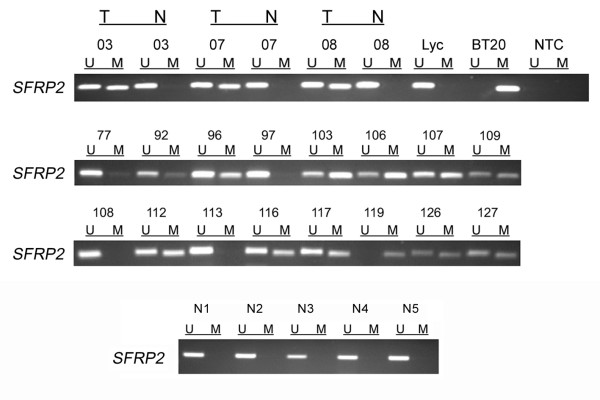
***SFRP2 *methylation analysis of primary breast cancer specimens**. MSP was performed on bisulfite-treated DNA from primary invasive breast cancer tissues. MSP results from 19 representative patient samples are shown. DNA bands in lanes labeled with U indicate PCR products amplified with primers recognizing the unmethylated *SFRP2 *promoter sequence. DNA bands in lanes labeled with M represent amplified products with methylation-specific primers. In addition, five representative normal cancer-unrelated breast tissues (N1 – N5) are shown. DNA from the breast cancer cell line BT20 and lymphocyte DNA from a healthy donor (Lyc) served as positive controls for MSP. NTC designates the no template control; T indicates tumor tissue; N indicates normal breast tissue.

In order to answer the question whether *SFRP2 *promoter methylation occurs in primary breast carcinoma as well we analyzed 199 mammary tumor samples by MSP. For 20 breast tumors corresponding tissues of histological normal breast epithelium were available and analyzed in parallel. Representative results are shown in Figure 3. In total, 165 of 199 tumor samples (83%) showed *SFRP2 *promoter methylation as a PCR product could be amplified with methylation-specific primers (*e.g*. #103 in Figure 3), and 34 of 199 tumors (17%) showed no evidence of promoter methylation since exclusive amplification signals were obtained with primers specific to unmethylated DNA (*e.g*. #108 in Figure 3). None of the 20 matched normal breast tissue samples showed a methylation signal (*e.g*. #03 in Figure 3). Tumor samples generally exhibited also unmethylated promoter sequences due to possible contamination with small amounts of stromal and endothelial cells, as has also been described by Suzuki *et al*. [19]. To further confirm that *SFRP2 *promoter methylation in breast cancer is restricted to malignant tissue, we analyzed 17 cancer-unrelated normal breast samples. Again, none of these normal breast tissues (*e.g*. Figure 3) harbored detectable *SFRP2 *methylation.

### Correlation of *SFRP2 *promoter methylation and *SFRP2 *mRNA expression in breast cancer cell lines

**Figure 4:**
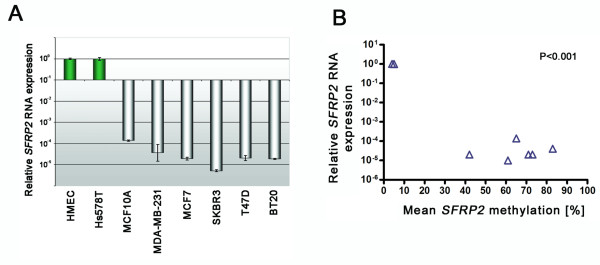
**Correlation of *SFRP2 *promoter methylation and loss of *SFRP2 *mRNA expression in breast cell lines**. (A) Realtime PCR of eight breast cell lines revealed abundant *SFRP2 *mRNA expression in benign HMEC and malignant Hs578T cells (green bars), whereas in all other investigated malignant breast cell lines *SFRP2 *mRNA expression was substantially reduced (grey bars). (B) Plotting of *SFRP2 *mRNA expression from the same cell lines (Y-axis) against each mean *SFRP2 *promoter methylation value (X-axis) reveals a significant correlation between loss of RNA expression and *SFRP2 *promoter methylation (*P *= 0.001; Pearson's correlation coefficient r = -0.9241).

In order to investigate the association of *SFRP2 *promoter methylation with transcriptional silencing in a quantitative manner we assessed in parallel *SFRP2 *mRNA expression by realtime PCR and *SFRP2 *methylation by quantitative Pyrosequencing in breast cell lines. The technical controls for Pyrosequencing revealed median methylation of 4% (unmethylated control) and 90% (methylated control) in the 22 sequential CpG sites, by this defining the detection limits of this assay. HMEC cells exhibited abundant *SFRP2 *mRNA expression (Δcycle threshold (C_T_) *GAPDH*:*SFRP2 *= 5.1) (Figure 4A) together with median *SFRP2 *methylation of 4%, in contrast to *e.g*. BT20 cells which exhibited mean methylation of 83% together with absence of *SFRP2 *mRNA expression (ΔC_T _*GAPDH*:*SFRP2 *= 19.9). Median *SFRP2 *methylation for Hs578T, MCF10A, MCF7, MDA-MB-231, SKBR3 and T47D was 5%, 73%, 73%, 71%, 61% and 42%, respectively. A direct comparison of *SFRP2* mRNA expression and *SFRP2* promoter methylation indicates that in breast cell lines *SFRP2 *methylation is correlated with loss of *SFRP2 *mRNA expression (*P *< 0.001; Figure 4B). Thus, when applying an empiric cut-off of > 5% to discriminate between *SFRP2 *methylation and non-methylation, all semi-quantitative results for *SFRP2 *methylation in breast cell lines obtained by MSP could be confirmed by the quantitative Pyrosequencing assay.

### Differential expression of SFRP2 protein in primary breast cancer

**Figure 5:**
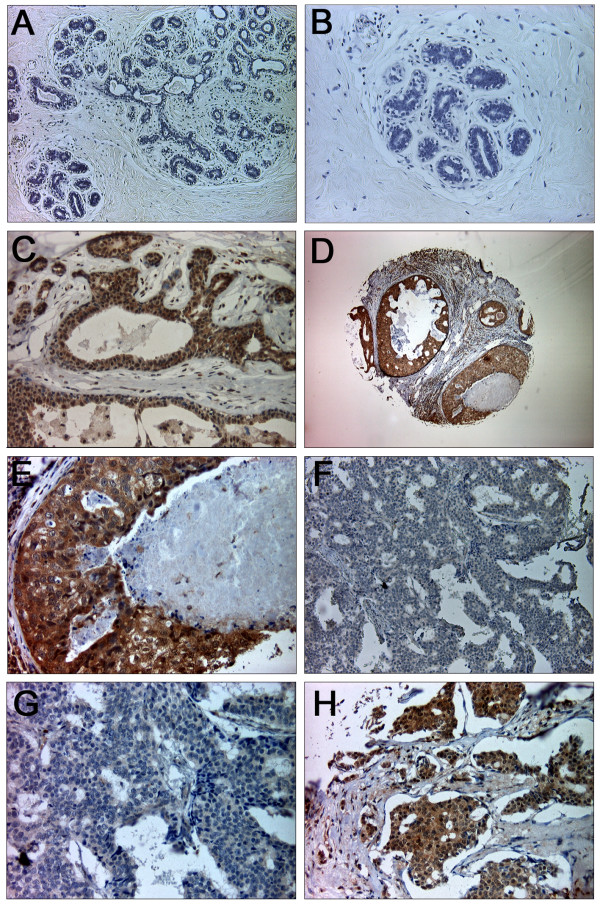
**SFRP2 protein expression in normal and malignant breast tissues**. (A) In the negative control showing normal breast tissue the primary SFRP2 antibody was omitted. (B) Scale-up of negative control shown in A. (C) Strong SFRP2 expression in ducts and lobules of normal breast tissue (IRS = 12). SFRP2 expression is abundant in epithelial cells, while there is only weak expression in stromal cells. (D) Very abundant SFRP2 expression in ductal carcinoma *in situ *of the breast. (E) Scale-up of specimen shown in D. (F) High-grade tumor exhibiting substantial loss of SFRP2 expression (IRS = 0). (G) Scale-up of specimen shown in F. (H) High-grade breast carcinoma with intensive cytoplasmic SFRP2 staining (IRS = 12). Original magnifications: A, F = 100×; B, C, G, H = 200×; D = 40×; E = 400×.

Immunohistochemical analysis was used to investigate SFRP2 protein expression in normal and malignant breast tissue. A total of 125 informative breast cancer cases, four DCIS and 10 normal breast tissues were analyzed. Intensity of immunohistochemical staining was evaluated using a semi-quantitative IRS [39]. SFRP2 protein was clearly detectable in 90% (9/10) of normal breast tissue samples analyzed, as defined by an IRS ≥ 8 (Figure 5C). The mean expression was determined to be IRS = 7.5 (range 3–8; standard deviation (SD) ± 1.6), and median expression to be IRS = 8. Expression was predominantly localized in luminal and basal epithelial cells of the normal breast while weak expression was detectable in adjacent stromal cells. In four DCIS, SFRP2 expression was slightly reduced (mean IRS = 6.5; range 4–8; SD ± 1.9; median IRS = 7; Figure 5D, E) with one sample showing strong reduction (25%, IRS = 4). However, the mean expression in invasive breast carcinomas was determined to be IRS = 4.6 (range 0–8; SD ± 2.1) and median expression to be IRS = 4. Invasive breast carcinomas showed strongly reduced or complete loss (IRS ≤ 4) of SFRP2 expression in 74% (93/125) of cases (Figure 5F, G). The SFRP2 expression difference between tumors and normal breast tissues was statistically significant (*P *= 0.001).

### Correlation of SFRP2 expression and *SFRP2 *methylation with clinicopathological parameters and patient survival

**Figure 6:**
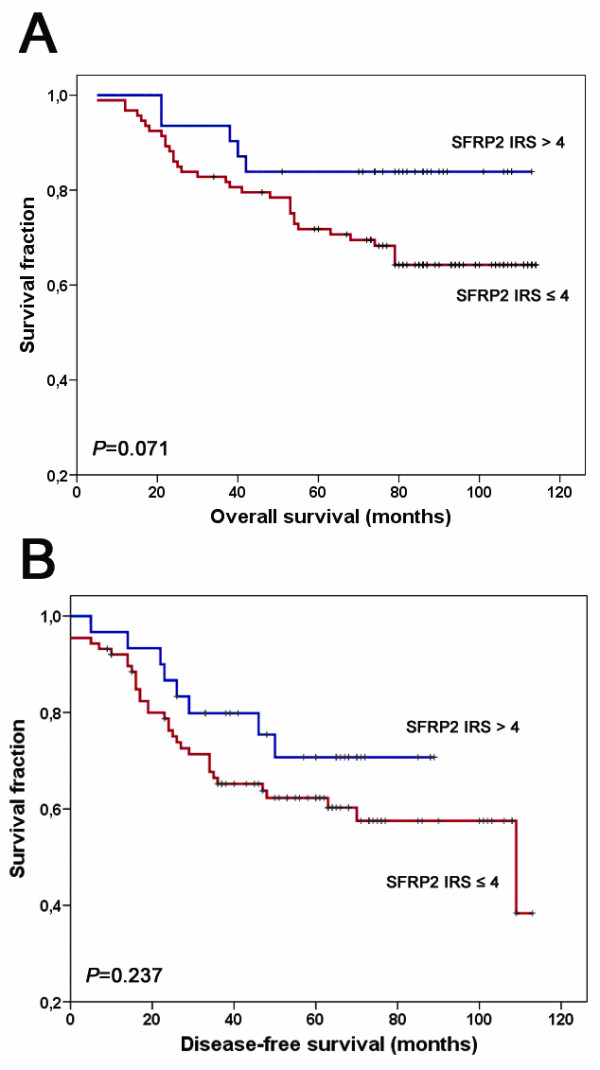
**Loss of SFRP2 protein expression in correlation to patient survival**. (A) Univariate Kaplan-Meier analysis of patient overall survival stratified between SFRP2 expresser (IRS > 4, blue line) and SFRP2 non-expresser (IRS ≤ 4, red line). A weak association of loss of SFRP2 protein expression with unfavorable outcome could be detected (*P *= 0.071, two-sided log-rank test). (B) Univariate Kaplan-Meier analysis regarding disease-free patient survival. The visual impression of a clinical impact of SFRP2 protein expression loss on breast tumor recurrence was statistically not significant (*P *= 0.237, two-sided log-rank test). Vertical tick marks represent censored patients.

Clinicopathological characteristics of breast cancer patients were first correlated with SFRP2 protein expression for descriptive data analysis (Table 1). Loss of SFRP2 expression in tumor tissue (IRS ≤ 4) was not associated with age at diagnosis, tumor size, histological grade, histological type, estrogen/progesterone receptor status, Her2 status or expression of p53. A prevalence of more abundant SFRP2 expression was detected in node negative breast tumors (*P *= 0.033). In univariate survival analysis using log-rank test, loss of SFRP2 protein expression was not associated with DFS (*P *= 0.237), but a weak trend was detected towards reduced OS (*P *= 0.071) (Figure 6). *SFRP2 *promoter methylation in breast carcinomas was not associated with age at diagnosis, tumor size, lymph node status, histological grade, histological type, or estrogen/progesterone receptor status (Table 2). In univariate survival analysis, lymph node status, histological grade and histological type were significantly associated with DFS; lymph node status and histological grade were significantly associated with OS (Table 3). However, *SFRP2 *methylation was neither associated with DFS (*P *= 0.192) nor with OS intervals (*P *= 0.686).

**Table 1:** Clinicopathological and immunohistochemical factors in relation to SFRP2 immunoreactivity

	**SFRP2 immunoreactivity**				
	
**Variable**	**n**^a^	**IRS 0 – 4 (%)**	**IRS > 4 (%)**	**R**^d^	***P***^e^
**Total**	125	93 (74)	32 (26)		

***Clinicopathological factors***					

Age at diagnosis (median: 57 years; range 29 – 82 years)					
< 60 years	69	49 (71)	20 (29)	-0.103	0.255
≥ 60 years	55	44 (80)	11 (20)		

Tumor size^b^					
pT1	42	29 (69)	13 (31)	-0.110	0.226
pT2 – 4	81	64 (79)	17 (21)		

Lymph node status^b^					
pN0	62	41 (66)	21 (34)	-0.194	**0.033**
pN1 – 3	59	49 (83)	10 (17)		

Histological grade					
G1 – G2	67	49 (73)	18 (27)	-0.047	0.606
G3	57	44 (77)	13 (23)		

Histological type					
ductal	98	73 (75)	25 (26)	0.024	0.796
lobular	9	9 (100)	0 (0)		
other	14	9 (64)	5 (36)		

***Immunohistochemistry***					

Estrogen receptor status					
negative (IRS^c ^0 – 2)	28	20 (71)	8 (29)	-0.079	0.424
positive (IRS 3 – 12)	76	60 (79)	16 (21)		

Progesterone receptor status					
negative (IRS^c ^0 – 2)	72	55 (76)	17 (24)	0.048	0.611
positive (IRS 3 – 12)	43	31 (72)	12 (28)		

Her2					
negative (0, 1+)	91	70 (77)	21 (23)	0.110	0.238
positive (2+, 3+)	26	17 (65)	9 (35)		

p53					
negative (< 5%)	70	53 (76)	17 (24)	0.033	0.724
positive (≥ 5%)	44	32 (73)	12 (27)		

^a^Only female patients with primary, unilateral, invasive breast cancer were included. ^b^According to UICC: TNM Classification of Malignant Tumours [38]. ^c^IRS = immunoreactivity score according to Remmele and Stegner [40]. ^d^Pearson's correlation coefficient. ^e^Fisher's exact test (two-sided). Significant *P*-values are marked in bold face. Percentages may not sum to 100 due to rounding.

**Table 2:** Correlation analysis of *SFRP2 *promoter methylation with clinicopathological and immunohistochemical patient characteristics

	***SFRP2 *promoter**				
	
**Variable**	**n**^a^	**Unmethylated (%)**	**Methylated (%)**	**R**^b^	***P***^c^
**Total**	199	34 (17)	165 (83)		

***Clinicopathological factors***					

Age at diagnosis					
< 60 years	114	24 (21)	90 (79)	0.122	0.091
≥ 60 years	85	10 (12)	75 (88)		

Tumor size^d^					
pT1	70	11 (16)	59 (84)	-0.013	1.000
pT2 – 4	114	19 (17)	95 (83)		

Lymph node status^d^					
pN0	89	19 (21)	70 (79)	0.129	0.106
pN1 – 3	85	10 (12)	75 (88)		

Histological grade					
G1 – G2	109	13 (12)	96 (88)	-0.139	0.069
G3	76	17 (22)	59 (78)		

Histological type					
ductal	155	26 (17)	129 (83)	-0.057	0.434
lobular	24	1 (4)	23 (96)		
other	14	5 (36)	9 (64)		

***Immunohistochemistry***					

Estrogen receptor					
negative (IRS^e ^0 – 2)	64	13 (20)	51 (80)	0.084	0.295
positive (IRS 3 – 12)	123	17 (14)	106 (86)		

Progesterone receptor					
negative (IRS^e ^0 – 2)	70	14 (20)	56 (80)	0.083	0.304
positive (IRS 3 – 12)	117	16 (14)	101 (86)		

^a^Only female patients with primary, unilateral, invasive breast cancer were included. ^b^Pearson's correlation coefficient. ^c^Fisher's exact test (two-sided). ^d^According to UICC: TNM Classification of Malignant Tumours [38]. ^e^IRS = immunoreactivity score [40]. Significant *P*-values marked in bold face.

**Table 3:** Univariate survival analysis of clinicopathological and immunohistochemical parameters with *SFRP2 *promoter methylation

	**Disease-free survival (DFS)**			**Overall survival (OS)**		
	
**Variable**	**n**^a^	**Events**	***P***^b^	**n**^a^	**Events**	***P***^b^
***Clinicopathological factors***						

Age at diagnosis						
< 60 years	84	33	0.391	83	15	0.414
≥ 60 years	52	18		53	13	

Tumor size^c^						
pT1	51	16	0.059	51	8	0.165
pT2 – 4	82	35		82	20	

Lymph node status^c^						
pN0	59	15	**0.008**	59	7	**0.026**
pN1 – 3	66	31		66	18	

Histological grade						
G1 – G2	72	19	**0.003**	73	7	**0.001**
G3	61	32		61	21	

Histological type						
ductal	109	35	**0.009**	109	25	0.622
lobular	18	9		18	2	
other	9	7		9	1	

***Immunohistochemistry***						

Estrogen receptor						
negative (IRS^d ^0 – 2)	47	16	0.644	46	12	0.118
positive (IRS 3 – 12)	84	35		85	16	

Progesterone receptor						
negative (IRS^d ^0 – 2)	44	19	0.318	45	12	0.093
positive (IRS 3 – 12)	87	32		86	16	

***SFRP2 promoter***						

unmethylated	23	5	0.192	23	5	0.686
methylated	113	45		113	23	

^a^Only female patients with primary, unilateral, invasive breast cancer were included. ^b^Univariate log-rank test (two-sided). ^c^According to UICC: TNM Classification of Malignant Tumours [38]. ^d^IRS = immunoreactivity score [40]. Significant *P*-values marked in bold face.

### SFRP2 inhibits proliferation in breast cell lines

**Figure 7:**
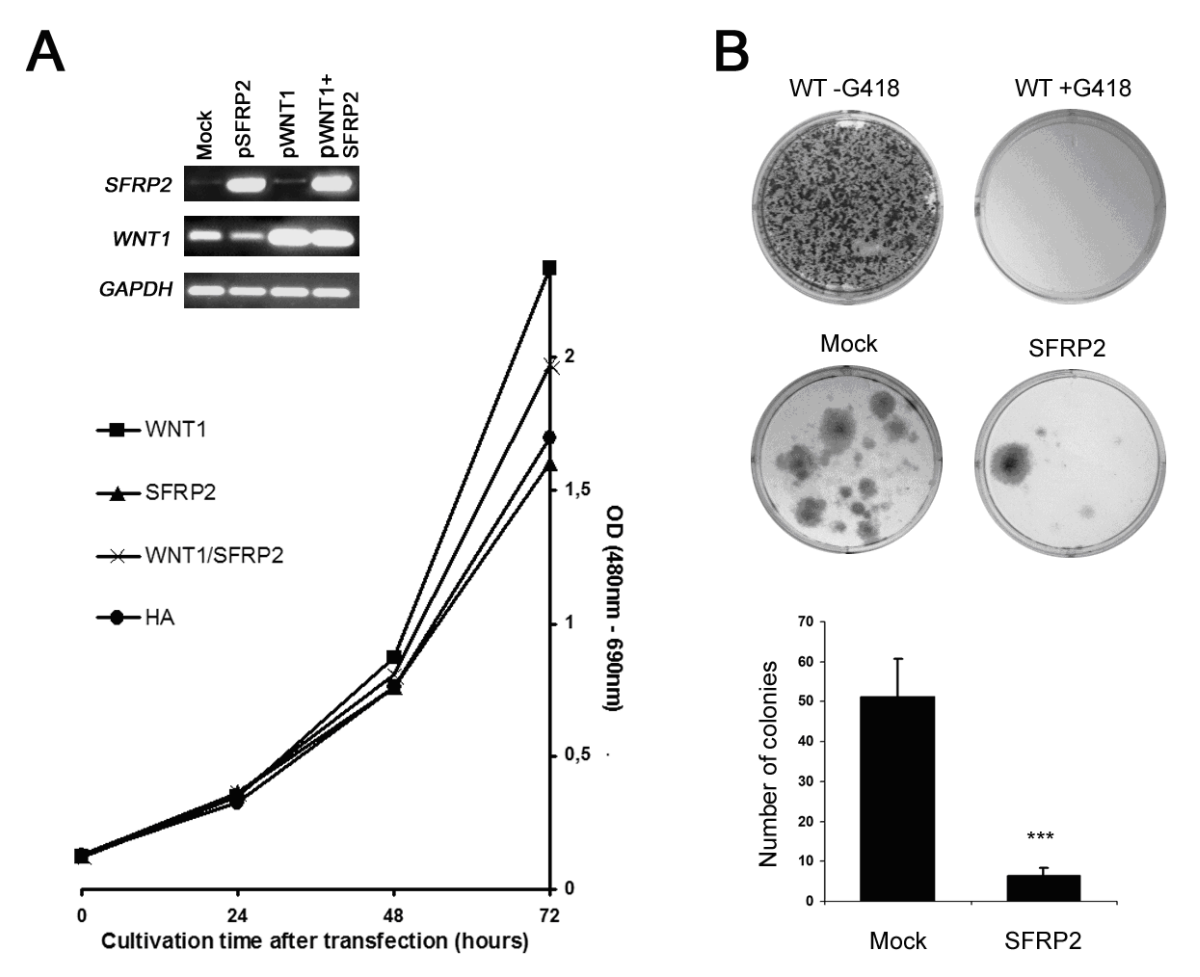
**SFRP2 inhibits proliferation in breast epithelial cells**. (A) MCF10A cells transfected with empty vector (Mock), *SFRP2*-expression vector, *WNT1*-expression vector or a combination of *SFRP2*+*WNT1*-expression vectors were subjected to XTT proliferation assays. At three subsequent time points, optical densities (OD) of the supernatants were measured. RNA Expression of the respective genes was surveyed in parallel 4 days post transfection. *GAPDH *served as cDNA loading control. *SFRP2 *was able to reduce a proliferative effect mediated by *WNT1 *overexpression, whereas the inhibiting effect of *SFRP2 *on cells not stimulated by *WNT1 *was only marginal (HA). (B) Long-term inhibiting effects of SFRP2 on the ability to form colonies in MCF10A cells. Cells were either transfected with empty vector (Mock) or with *SFRP2*-expression vector, and after 3 weeks of selective pressure (antibiotic G418) fixed, stained and photographed. Non-transfected wild type cells (WT) are shown as controls with (+) and without (-) the application of G418. The difference in colony numbers between mock-transfected and *SFRP2*-transfected cells is significant (*P *= 0.045; two-tailed Mann-Whitney U-test).

Finally, we asked whether SFRP2 influences proliferation rates in breast cell lines. For gain-of-function experiments we chose mammary MCF10A cells since these cells were found to lack *SFRP2* mRNA expression. As shown in Figure 7A, *WNT1 *overexpressing MCF10A cells notably increased proliferation compared to cells mock-transfected with empty vector. The equimolar co-transfection with *SFRP2*resulted in a decreased proliferation rate as compared to *WNT1 *alone. Furthermore, *SFRP2*-transfectants revealed a slightly reduced proliferation rate compared to cells containing empty vector. In order to support these findings we performed a colony formation assay and selected transfected clones for three weeks by the antibiotic G418. Representative results are shown in Figure 7B. Controls assured the feasibility of this assay, showing that wild type cells without G418 abundantly form colonies, whereas in the presence of G418 wild type cells fail to survive. *SFRP2*-transfected cells exhibit a reduced number of colonies as compared to mock-transfected cells. The difference in colony numbers from three independent experiments was statistically significant (*P *= 0.045, two-tailed Mann-Whitney U-test).

## Discussion

Aberrant methylation of CpG islands in gene promoters has been ascertained as a primary mechanism for the inactivation of tumor suppressor genes in human malignancies, including colon and breast cancer (for review see [3]). Clinically, the identification of genes that are prone to abnormal methylation and consequently become downregulated is of critical importance since this is considered to provide a good source of novel tumor biomarkers [52] and potential targets for chemotherapeutics [53,54]. The family of SFRP genes, functionally acting as Wnt signaling inhibitors, was recently shown to be a common target of promoter hypermethylation in numerous tumor entities [19-26]. In human breast cancer, we have previously shown that the *SFRP1 *and *SFRP5 *promoter is epigenetically silenced in 61% and 73% of invasive breast carcinomas, respectively, each of which was associated with unfavorable patient prognosis [27,28]. We here demonstrate that promoter methylation of *SFRP2 *is a further tumor-related alteration in human breast cancer occurring with even higher incidence.

Initiating our study, we found that many breast cancer cell lines revealed abolished *SFRP2 *expression presumably due to methylation of the *SFRP2 *promoter, since those cell lines lacking *SFRP2 *methylation abundantly expressed *SFRP2 *mRNA, whereas all cell lines lacking *SFRP2 *expression harbored *SFRP2 *promoter methylation. A direct coherence between promoter methylation and loss of RNA expression was shown by a combined DAC/TSA treatment of breast cancer cell lines, demonstrating that the *SFRP2 *gene was effectively demethylated and re-expressed after the treatment. Furthermore, those cell lines revealed a significant reduction of cyclin D1 expression, suggesting reactivation of anti-proliferative genes, of which *SFRP2 *is supposed to be a member [19,23,24]. Interestingly, in partially methylated SKBR3 and T47D cells the sole inhibition of histone deacetylases by TSA led to restoration of some *SFRP2 *expression, indicating that besides DNA methylation in these cells further reversible chromatin repressing histone modifications may exist.

Since cell lines may acquire *de novo *genetic and epigenetic lesions during cultivation [55,56] it is mandatory to investigate such aberrations in primary tissues as well. To this end we analyzed *SFRP2 *promoter methylation in 199 infiltrating breast carcinomas by MSP. We found a high-frequent incidence of *SFRP2 *methylation in the tumors (83%), confirming the recent results from Suzuki *et al*. [37], who reported of *SFRP2 *methylation in 60 of 78 (77%) primary breast carcinomas. Importantly, *SFRP2 *methylation was independent of relevant clinicopathological factors, thus being unlikely related to disease stage or a molecular breast cancer subtype. *SFRP2 *methylation was equally prevalent in small sized (pT1) and in larger sized (pT2-4) breast carcinomas, suggesting it occurs as early epigenetic aberration in breast tumorigenesis with no further increase in methylation frequency during tumor progression. Whether *SFRP2 *methylation is already present in benign and earlier premalignant lesions such as atypical hyperplasia and carcinoma *in situ*, like it was recently reported for the 14-3-3-σ gene [9], will be of particular importance in regard of early breast tumor detection. Yet, this remains to be determined in a future study.

Interestingly, Suzuki *et al*. [37] reported that a certain number of cancer-related normal breast tissues also showed weak *SFRP2 *methylation in their study, whereas in our study none of the normal breast tissues harbored a methylated *SFRP2 *promoter, irrespective of whether the tissue was taken from matched cancer-related or unmatched cancer-unrelated specimens. Given that no contaminating tumor cells had been present in their normal breast tissues this might be due to different locations of the recruited tissues (*i.e*. distance to the tumor margin), and may address a phenomenon that in cancer research is currently being discussed as "field defect" [57,58]. Evidence of such field defect in breast cancer was brought up by Yan *et al*. [59] showing that *RASSF1A *promoter methylation in breast carcinoma may progressively diffuse outwards to surrounding normal tissue, establishing a sphere of methylation gradient around the primary tumor. Recently, such gradient was also detected for *RUNX3 *methylation [60], which together with *RASSF1A *methylation is among the earliest carcinogenetic events in breast tumor transformation. *SFRP2 *methylation may be implicated in such field defect in breast cancer, yet dense methylation of the *SFRP2 *promoter was restricted to carcinoma in our study, and thus it may display important clinical specificity. In bladder cancer, *SFRP2 *methylation was shown to represent an independent predictor of malignancy, although in multivariate logistic regression analysis it was not a reliable biomarker because of a limited sensitivity/specificity due to some extent of methylation in normal bladder mucosa [22]. In contrast, in faecal DNA *SFRP2 *methylation was proven to be a highly promising screening marker for colorectal cancer [34], even potent to detect early lesions like adenoma, aberrant crypt foci [35] and colorectal polyps [36] due to the absence of *SFRP2 *methylation in normal colonic mucosa. In breast cancer, the accurate specificity and sensitivity of *SFRP2 *methylation remains to be determined by quantitative methods in a future study, for instance by qMSP (MethyLight) [61] or the Pyrosequencing technique [62], integrating receiver-operating characteristic (ROC) curve analyses. This may potentially lead to a valuable early tumor detection marker that will ideally be assessable in patients' body fluids like blood serum, plasma or nipple aspirate.

Contrasting the view that *SFRP2 *acts as a tumor suppressor gene, Lee and co-workers [42,63] suggested that *SFRP2 *exhibits rather an oncogenic property in breast tissue since this group detected strong upregulation of SFRP2 protein in canine mammary tumors relative to normal canine breast tissues. In addition, *SFRP2 *overexpression in a human breast cancer cell line (MCF7) inhibited apoptosis following UV light exposure, while increasing cell-substrate adhesion capacity [64]. It is worthy to note that these experiments were carried out with a canine homologue of *SFRP2 *cDNA. However, five lines of evidence propose a tumor suppressive role of *SFRP2 *in human breast carcinogenesis: (1.) Our and another independent study [37] demonstrate that *SFRP2 *is very frequently targeted by promoter methylation in human breast carcinomas as compared to normal human breast tissues, disposing breast cancer to the large number of human tumor entities for which *SFRP2 *methylation has already been described. (2.) We found a strong correlation and functional association of *SFRP2 *methylation with loss of *SFRP2* mRNA expression in breast cell lines. (3.) Our study reveals a common SFRP2 protein loss in human breast carcinomas with comparable frequency to promoter methylation, notably by applying the identical SFRP2-antibody that was used for the study of canine mammary tumors. (4.) We detected a weak trend towards adverse clinical patient outcome in case of SFRP2 protein expression loss. (5.) Functional analyses in human breast [37], gastric [23,24] and colorectal cancer cell lines [19] revealed a pro-apoptotic and anti-proliferative capacity of (human) SFRP2 associated with the ability to inhibit activated Wnt signaling, altogether supporting a tumor suppressive rather than an oncogenic function of this gene. These discrepancies to canine mammary tumors may reflect subtle distinctions in the function of structurally related molecules, or alternative activities of molecules when expressed in different contexts and organisms. Furthermore, it emphasizes that study results of *SFRP2 *from canine breast cancer models may not be generally transferable to human breast carcinogenesis. In conclusion, *SFRP2 *may represent a candidate class II tumor suppressor gene whose altered expression is caused by epigenetic changes (class II) rather than by mutation (class I) [65]. Class II tumor suppressor genes are particularly interesting drug targets since reversing the block of their gene expression, *e.g*. by DNA methyltransferase (DNMT) inhibitors or histone deacetylase (HDAC) inhibitors could lead to tumor regression. Furthermore such a treatment could be appropriate to eliminate minimal residual cancer disease after surgical resection of the tumor.

Summarizing, our data demonstrate that *SFRP2 *is a frequent target of epigenetic inactivation in human breast cancer leading to downregulation of *SFRP2 *expression in mammary tumors. Loss of *SFRP2 *expression confers a growth advantage to mammary cells, likely due its ability to inhibit oncogenic Wnt signaling. Altogether, our data support the proposed tumor suppressive function of *SFRP2 *in normal breast tissue. The high incidence and the putative specificity of this epimutation may qualify *SFRP2 *methylation as potential candidate in a screening marker panel for the early detection of human breast cancer.

## Conclusion

Our study on *SFRP2 *in human breast cancer leads to the following conclusions: *SFRP2 *expression is very frequently downregulated in breast cancer due to promoter methylation, thus conferring growth advantage to neoplastic mammary cells. Therefore, *SFRP2 *may be assigned a class II tumor suppressor gene in normal breast tissue, whose block of expression could be reversed by DNA demethylating (DNMT inhibitors) and histone reacetylating (HDAC inhibitors) drugs. In contrast to an adverse prognostic value of *SFRP1 *or *SFRP5 *methylation in breast cancer, failure of *SFRP2 *methylation as a prognostic biomarker may be explained by redundant functions of these closely related SFRP molecules. Alternatively, this failure could be explained by the likely involvement of *SFRP2 *methylation in the early steps of breast carcinogenesis, rather than being implicated in the development of prognostically adverse tumor subtypes. Nevertheless, *SFRP2 *methylation may be potentially useful as a molecular tumor biomarker in a DNA methylation biomarker based screening assay, as it may display high clinical sensitivity and specificity in detecting breast cancer cells.

## Competing interests

The authors declare that they have no competing interests.

## Authors' contributions

JV participated in the design of the study, carried out the RNA expression and methylation analyses, immunohistochemical studies, *in vitro *experiments, statistical analysis, and wrote the manuscript. EN performed realtime expression analysis, assisted in Pyrosequencing and data interpretation, and critically revised the manuscript. NB participated in immunohistochemical analysis and data interpretation, and critically revised the manuscript. EJ designed and optimized the *SFRP2 *Pyrosequencing assay, and critically revised the manuscript. AH participated in collection of clinical data, performed data interpretation, and critically revised the manuscript. RK participated in the design and coordination of the study, and critically revised the manuscript. ED planned and coordinated the study, and critically revised the manuscript. All authors have read and approved the final version of the manuscript.

## Supplementary Material

Additional file 1 **Clinicopathological and immunohistochemical characteristics of primary invasive breast carcinomas (n = 199).** The data provided represent the relevant clinicopathological and immunohistochemical patient characteristics used in *SFRP2 *methylation analysis. Click here for file

Additional file 2 **Primer sequences and cycle conditions used in this study.** This table provides oligonucleotide primer sequences and PCR cycle conditions that were used throughout this study. Click here for file
