# Supplementary material for: Promoter hypermethylation of the SFRP2 gene is a high-frequent alteration and tumor-specific epigenetic marker in human breast cancer
Source: Mol Cancer. 2008 Nov 6;7:83. doi: 10.1186/1476-4598-7-83 (PMC2613402; doi:10.1186/1476-4598-7-83)
Supplement: Additional file 1 — Clinicopathological and immunohistochemical characteristics of primary invasive breast carcinomas (n = 199). The data provided represent the relevant clinicopathological and immunohistochemical patient characteristics used in SFRP2 methylation analysis. [file 1476-4598-7-83-S1.doc]

**Additional file 1: Clinicopathological and immunohistochemical characteristics of primary invasive breast carcinomas (n=199)**

| **Variable** | **Categorization** | **n**^a^ | **%** |
| --- | --- | --- | --- |
|
| ***Clinicopathological factors*** | | | |
| Age at diagnosis | | | |
| Median: 57 years (range 28-96 years) | | | |
|  | < 60 years | 114 | 57.2 |
|  | ≥ 60 years | 85 | 42.7 |
| Tumor size^b^ | | | |
|  | pT1 | 70 | 35.2 |
|  | pT2 | 88 | 44.2 |
|  | pT3 | 13 | 6.5 |
|  | pT4 | 13 | 6.5 |
|  | pTx | 15 | 7.5 |
| Lymph node status^b^ | | | |
|  | pN0 | 89 | 44.7 |
|  | pN1 - 3 | 85 | 42.7 |
|  | pNx | 25 | 12.6 |
| Histological grade | | | |
|  | G1 | 15 | 7.5 |
|  | G2 | 94 | 47.2 |
|  | G3 | 76 | 38.2 |
|  | x^c^ | 14 | 7.0 |
| Histological type | | | |
|  | ductal | 155 | 77.9 |
|  | lobular | 24 | 12.1 |
|  | other | 14 | 7.0 |
|  | x^c^ | 6 | 3.0 |
| ***Immunohistochemistry (IHC)*** | | | |
| Estrogen receptor | | | |
|  | negative (IRS^d^ 0 - 2) | 64 | 32.2 |
|  | positive (IRS 3 - 12) | 123 | 61.8 |
|  | x^c^ | 12 | 6.0 |
| Progesterone receptor | | | |
|  | negative (IRS^d^ 0 - 2) | 70 | 35.2 |
|  | positive (IRS 3 - 12) | 117 | 58.8 |
|  | x^c^ | 12 | 6.0 |

^a^Only female patients with primary, unilateral, invasive breast cancer were included. ^b^According to UICC: TNM Classification of Malignant Tumours [38]. ^c^x = status unknown. ^d^IRS = immunoreactivity score [40]. Percentages may not sum to 100 due to rounding.
