# Supplementary material for: Promoter hypermethylation of the SFRP2 gene is a high-frequent alteration and tumor-specific epigenetic marker in human breast cancer
Source: Mol Cancer. 2008 Nov 6;7:83. doi: 10.1186/1476-4598-7-83 (PMC2613402; doi:10.1186/1476-4598-7-83)
Supplement: Additional file 2 — Primer sequences and cycle conditions used in this study. This table provides oligonucleotide primer sequences and PCR cycle conditions that were used throughout this study. [file 1476-4598-7-83-S2.doc]

**Additional file 2: Primer sequences and cycle conditions used in this study**

|  | **Sequence (5' → 3')** | **T_A_ (ºC)** | **bp** |
| --- | --- | --- | --- |
| ***RT-PCR*** | | |  |
| GAPDH | Forward: TGGTCACCAGGGCTGCTT | 59 | 510 |
|  | Reverse: GTCTTCTGGGTGGCAGTGAT |
| SFRP2 | Forward: ATGATGATGACAACGACATAATG | 60 | 322 |
|  | Reverse: ATGCGCTTGAACTCTCTCTGC |
| WNT1 | Forward: CGAGTGCAAGTGGCAGTTC | 60 | 105 |
|  | Reverse: AACGCCGTTTCTCGACAG |
| ***Semi-quantitative realtime PCR*** | | | |
| GAPDH | Forward: GAAGGTGAAGGTCGGAGTCA | 60 | 108 |
|  | Reverse: TGGACTCCACGACGTACTCA |
| Cyclin D1 | Forward: CCGTCCATGCGGAAGATC | 60 | 98 |
|  | Reverse: ATGGCCAGCGGGAAGAC |
| SFRP2 | Forward: ATGATGATGACAACGACATAATG | 60 | 322 |
|  | Reverse: ATGCGCTTGAACTCTCTCTGC |
| ***Methylation-specific PCR*** | | | |
| SFRP2 unmethylated | Forward: TTTTGGGTTGGAGTTTTTTGGAGTTGTGT | 58 | 145 |
|  | Reverse: AACCCACTCTCTTCACTAAATACAACTCA |
| SFRP2 methylated | Forward: GGGTCGGAGTTTTTCGGAGTTGCGC | 58 | 138 |
|  | Reverse: CCGCTCTCTTCGCTAAATACGACTCG |

T_A_, annealing temperature; bp, product size (base pairs)
